# An ultrasound-based radiomics model for survival prediction in patients with endometrial cancer

**DOI:** 10.1007/s10396-023-01331-w

**Published:** 2023-06-13

**Authors:** Xiao-wan Huang, Jie Ding, Ru-ru Zheng, Jia-yao Ma, Meng-ting Cai, Martin Powell, Feng Lin, Yun-jun Yang, Chu Jin

**Affiliations:** 1 Department of Gynecology, The First Affiliated Hospital of Wenzhou Medical University, Wenzhou, 325000 People’s Republic of China; 2 Wenzhou Medical University Renji College, University Town, Chashan, Wenzhou, 325000 People’s Republic of China; 3 Department of Ultrasound Imaging, Yueqing Hospital of Wenzhou Medical University, Wenzhou, 325015 People’s Republic of China; 4 Department of Radiology, The First Affiliated Hospital of Wenzhou Medical University, Wenzhou, 325000 People’s Republic of China; 5 Nottingham Treatment Centre, Nottingham University Affiliated Hospital, Nottingham, NG7 2FT UK

**Keywords:** Radiomics features, Ultrasound, Endometrial cancer, Disease-free survival, Nomogram

## Abstract

**Purpose:**

To establish a nomogram integrating radiomics features based on ultrasound images and clinical parameters for predicting the prognosis of patients with endometrial cancer (EC).

**Materials and methods:**

A total of 175 eligible patients with ECs were enrolled in our study between January 2011 and April 2018. They were divided into a training cohort (*n* = 122) and a validation cohort (*n* = 53). Least absolute shrinkage and selection operator (LASSO) regression were applied for selection of key features, and a radiomics score (rad-score) was calculated. Patients were stratified into high risk and low-risk groups according to the rad-score. Univariate and multivariable COX regression analysis was used to select independent clinical parameters for disease-free survival (DFS). A combined model based on radiomics features and clinical parameters was ultimately established, and the performance was quantified with respect to discrimination and calibration.

**Results:**

Nine features were selected from 1130 features using LASSO regression in the training cohort, which yielded an area under the curve (AUC) of 0.823 and 0.792 to predict DFS in the training and validation cohorts, respectively. Patients with a higher rad-score were significantly associated with worse DFS. The combined nomogram, which was composed of clinically significant variables and radiomics features, showed a calibration and favorable performance for DFS prediction (AUC 0.893 and 0.885 in the training and validation cohorts, respectively).

**Conclusion:**

The combined nomogram could be used as a tool in predicting DFS and may assist individualized decision making and clinical treatment.

**Supplementary Information:**

The online version contains supplementary material available at 10.1007/s10396-023-01331-w.

## Introduction

As the leading gynecological malignancy in western countries, endometrial cancer (EC) ranks sixth among cancers in women worldwide [1, 2]. Most diagnoses are made at an early stage, thereby yielding favorable 5-year overall survival (OS), ranging from 74 to 91% [1, 3]. However, some patients may have dismal survival as a result of a delayed diagnosis. Metastasis is related to a worse outcome; the 5-year OS rate is 57–66% for stage III and only 20–26% for stage IV EC [4]. To date, the International Federation of Gynecology and Obstetrics (FIGO) staging system together with histological grade and type classification have been proven to be prognostic factors for EC [5]. Because of tumor heterogeneity, the prognosis of patients in the same stage varies. Therefore, difficulties still exist in the current EC classification system regarding prognosis estimation [6]. However, many clinical and pathological features [7, 8] have been suggested to predict the prognosis of EC patients, but the prognostic accuracy of these factors is still uncertain. Therefore, there is an urgent need to find a biomarker or a model that can accurately predict patients with high risk of adverse survival to guide postoperative management and establish follow-up protocols.

Ultrasound is recognized as a radiation-free, readily available, and easy-to-use method, and is currently the first-line imaging modality for the detection and diagnosis of EC. Radiomics is a methodology that converts digital medical images into high-dimensional mineable data [9, 10]. According to previous studies, radiomics have been successfully shown to correlate with clinical outcomes and biological endpoints across a wide range of solid tumors [11–14], including EC [15–17]. However, those studies mainly focused on computed tomography (CT) and magnetic resonance imaging (MRI), but CT is limited by exposure to severe radiation and MRI is limited by its cost, long examination length, and long waiting lists at most centers. More and more research has extended radiomics to US imaging, showing encouraging results [18–20]. But to our knowledge, there are no published studies that have investigated whether ultrasound-based radiomics can be used to estimate the prognosis of EC.

Therefore, in this study, we aimed to establish and validate a novel radiomics model based on US images that incorporates radiomics features and clinical parameters to estimate disease-free survival (DFS) in patients with EC.

## Materials and methods

### Patient data

This retrospective study was approved by the Ethics Committee of our hospital. A total of 612 patients who underwent surgery with pathologically confirmed EC between January 2011 and April 2018 were enrolled in the study. Clinical parameters, pretreatment ultrasound imaging data, pathologic results, and survival data were reviewed.

**Fig. 1:**
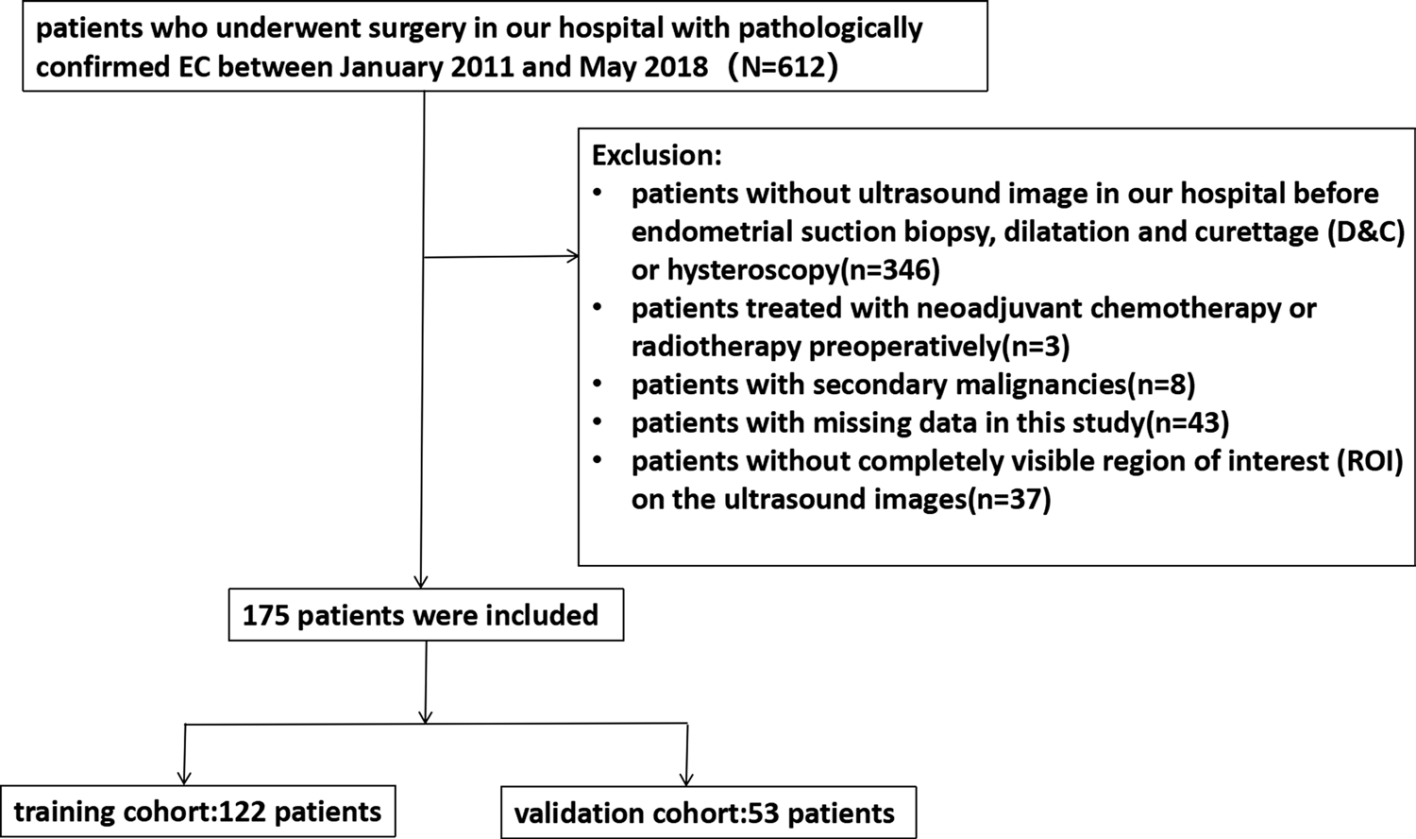
Recruitment pathway for patients in this study

All the patients met the following inclusion criteria: (1) pathologically confirmed EC; (2) underwent ultrasound at our hospital within 2 weeks before surgery; and (3) underwent total hysterectomy with bilateral salpingo-oophorectomy, with or without nodal staging (pelvic ± para-aortic lymphadenectomy). The exclusion criteria were as follows: (1) absence of ultrasound images at our hospital before endometrial suction biopsy, dilatation and curettage (D&C), or hysteroscopy; (2) underwent neoadjuvant chemotherapy or radiotherapy preoperatively; (3) presence of secondary malignancies; (4) missing data in this study; and (5) absence of completely visible region of interest (ROI) on ultrasound images. A total of 175 patients (mean age of 56.61 ± 8.68 years) were ultimately included in this study. The patient selection flowchart is shown in Fig. 1. The 175 eligible patients were then randomly divided into two independent cohorts at a ratio of 7:3: a training cohort (*n* = 122) and a validation cohort (*n* = 53).

### Clinical information

The following clinical parameters were retrieved from medical records: age, menopause status, body mass index (BMI, calculated as weight in kilograms divided by the square of height in meters), pathological type, differentiation, tumor size, FIGO stage, lymph node metastasis (LNM), depth of myometrial invasion (DMI), lymph-vascular space invasion (LVSI) status, CA-125, and rad-score. The endpoint of our study was DFS, which was defined as the time from surgery to either first EC recurrence or death. Recurrence was defined as a biopsy-proven tumor exhibiting EC or a lesion deemed suspicious on imaging, such as CT, MRI, and positron emission tomography-computed tomography. Survival information was obtained from medical records or by telephone follow-up, with the last follow-up being in April 2021.

### Image acquisition and tumor segmentation

All the procedures were performed according to the Image Biomarker Standardization Initiative (IBSI) standards. The ultrasound devices used at our hospital were Hitachi or Philips (IU22) units with linear probes (5–14 MHz). Static images were stored in a picture archiving and communication system (PACS) (DICOM format). Eight to twelve standard ultrasound images were recorded for each patient, and a representative image with the largest cross section of the tumor was selected. Since the images were selected using different ultrasound scanners, we normalized each image using resampling and gray-level discretization. The ultrasound images were two-dimensional images with a thickness of 1 mm; thus, we resampled the ultrasound images according to the voxel 1 × 1 × 1 mm. Thereafter, we performed intensity standardization and discretized the gray level in the range of 0–255.

For the target tumor, the tumor region of interest (ROI) covering the whole tumor was manually segmented by a radiologist with 15 years of experience in gynecological imaging using ITK-SNAP software (http://www.itksnap. org). To extract the radiomics features, the original images and the ROIs were imported into A.K. software (Artificial Intelligence Kit, version 3.0.0, GE Healthcare). Each patient’s original image and ROI were automatically matched one by one. And the fixed bin width (default = 10) was set before feature extraction.

### Radiomics feature extraction

A total of 1,130 radiomics features were extracted from the ROI, but not all of them were associated with the DFS in EC. A two-step feature selection method was applied for selection of key features that were significantly associated with DFS in the training cohort. First, univariate Cox regression was used to select radiomics features with a p value less than 0.05 in the training cohort, which were treated as significant prognostic features and selected as candidate features. Second, the least absolute shrinkage and selection operator (LASSO) regression algorithm was used for multivariate feature selection with nonzero coefficients from candidate features, with penalty parameter tuning conducted by tenfold cross-validation. The λ value that minimized standard deviation and maximum AUC of receiver operating characteristic (ROC) curves was selected as the optimal regularization parameter. The number of radiomics features was therefore automatically determined according to the λ value. Lastly, a formula for the rad-score was calculated by linear combination of the chosen features for further evaluation of the prediction model. Kaplan–Meier survival analysis was used to assess the stratification ability of rad-score, and comparisons were constructed through log-rank test. A heatmap was computed to analyze the correlation between the radiomics features and the independent clinical parameters for all patients. The numbers in the cells represent P values.

### Construction and validation of the models

Univariate Cox regression analysis was used to select the most significant clinical parameters, and variables with significance (*P* < 0.05) in the univariate analysis were quantified for being included in multivariate model to build the clinical model. To further evaluate whether the clinical parameters could improve the performance of the rad-score, a combined model incorporating the radiomics features and the clinical parameters was built for 3 year DFS prediction.

ROC analysis was applied to evaluate the prognostic performance of each model in predicting DFS. Each model’s discriminative performance was displayed as the AUC. Calibration was used to graphically assess agreement between the predicted outcome and the corresponding observed result (calibration curves). The diagnostic performance of the model for DFS prediction in EC was assessed with respect to AUC, sensitivity, specificity, and accuracy in both the training and validation cohorts.

A radiomics-based nomogram for individualized prognosis was created by incorporating the radiomics and clinical model. The total points accumulated for various variables corresponded to the prediction probability for a patient. Calibration curves were drawn to show how close the model’s estimation matched the observed prognosis.

### Statistical analysis

All the statistical analyses in this study were performed with R software (version 4.0.0, http://www.r-project.org) and SPSS22.0. A two-sided p value of less than 0.05 was considered statistically significant.

Differences in the clinicopathological parameters were evaluated using the Student’s t-test and chi-squared (*χ*2) test or Fisher’s exact test. LASSO-based feature selection with tenfold cross-validation was used for radiomics feature selection. Rad-scores were computed via a linear combination of the selected features weighted by each coefficient. Kaplan–Meier curves, nomogram drawings, and calibration curves were analyzed using the “rms” package. ROC curves were plotted with the “pROC” package. The AUC was calculated using the selected features or factors with a 95% confidence interval (CI). Accuracy, specificity, and sensitivity were calculated according to the cut-off value of the maximum Youden index.

## Results

### Study patients

A total of 175 EC patients were enrolled and then randomly divided into the training and validation cohorts at a ratio of 7:3, as shown in Fig. 1. Table 1 shows the clinical characteristics of the patients. No significant differences were found between the two cohorts (*p* > 0.05 for all). The median follow-up time was 49 months (range, 1–123 months).

**Table 1:** Patient characteristics in the training and validation cohorts

	Training cohort (*n* = 122)	Validation cohort (*n* = 53)	*P*
Age (years)			0.727
< 60	81	37	
≥ 60	41	16	
Menopause status			0.858
No	35	16	
Yes	87	37	
BMI	24.2009 ± 3.4156	24.9519 ± 3.5096	0.803
FIGO			0.186
IA	69	25	
IB	13	9	
II	22	6	
III–IV	18	13	
Pathological type			0.896
Endometrioid adenocarcinoma	100	43	
Non-endometrioid adenocarcinoma	22	10	
Differentiation			0.309
Low grade	29	17	
Middle grade	50	23	
High grade	43	13	
Tumor size			0.507
< 2 cm	18	10	
≥ 2 cm	104	43	
Lymph node metastasis(LNM)			0.063
Non-metastasis	113	44	
Metastasis	9	9	
Lymphovascular space invasion (LVSI)			0.150
Non-LVSI	109	43	
LVSI	13	10	
Depth of myometrial invasion (DMI)			0.579
< 1/2	91	37	
≥ 1/2	31	16	
CA125			0.678
< 35 U/ml	100	42	
≥ 35 U/ml	22	11	
Rad-score	0.02364	− 0.05443	0.337
Recurrence			0.638
Yes	26	13	
No	96	40	
Death			0.615
Yes	15	8	
No	107	45	

### Radiomics feature extraction and selection

**Fig. 2:**
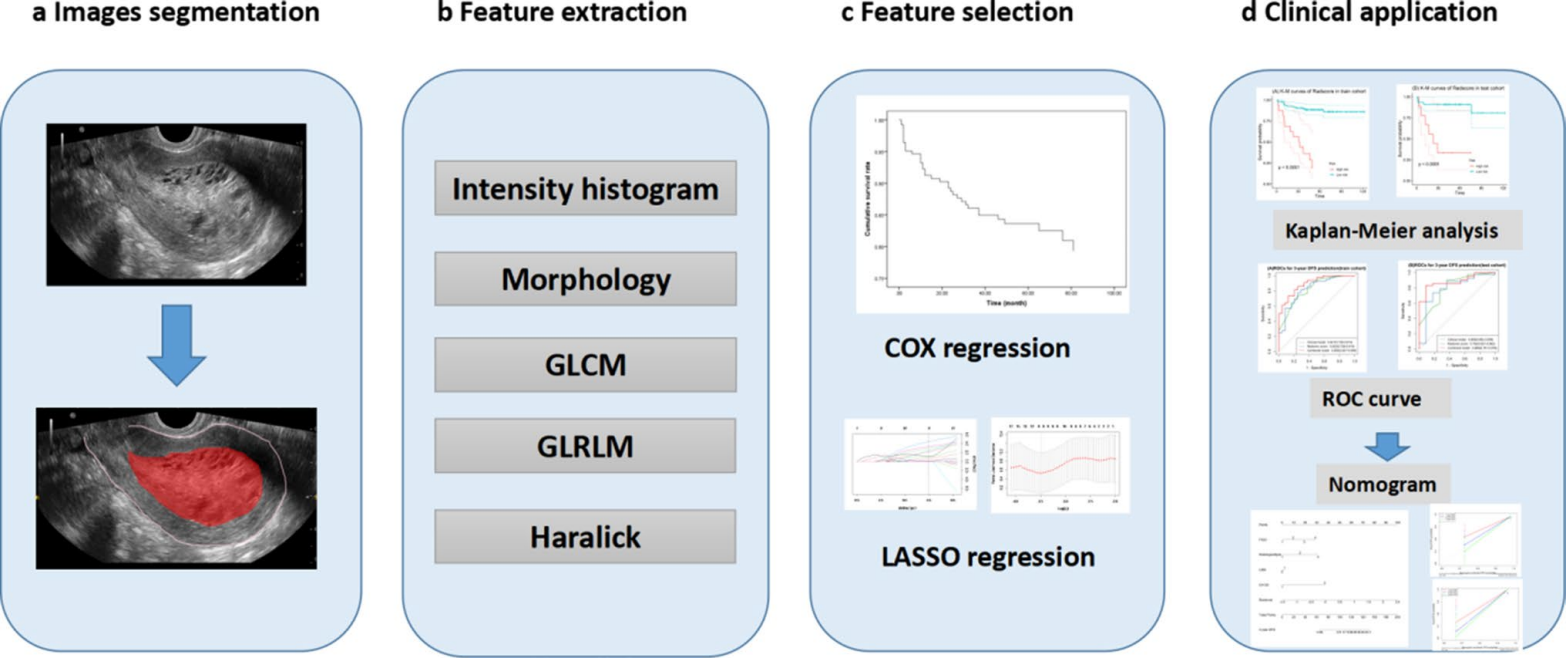
Flow diagram of radiomics model construction

**Fig. 3:**
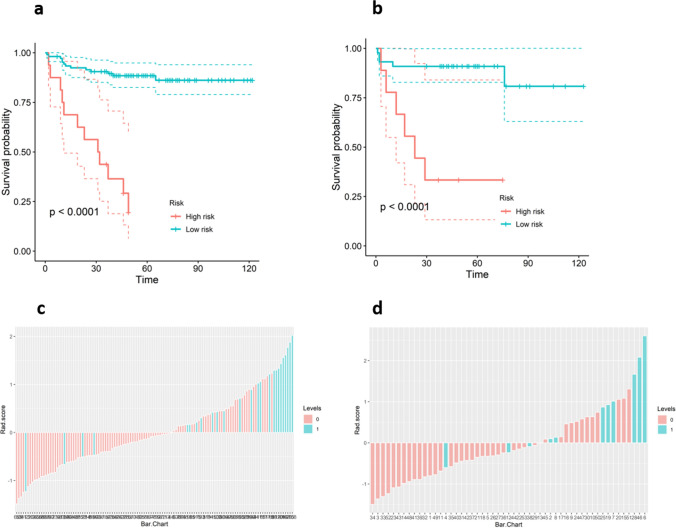
**a** Kaplan–Meier curves of the radiomics score in the training cohort. **b** Kaplan–Meier curves of the radiomics score in the validation cohort. **c** Radiomics score for each EC patient in the training cohort. **d** Radiomics score for each EC patient in the validation cohort

**Fig. 4:**
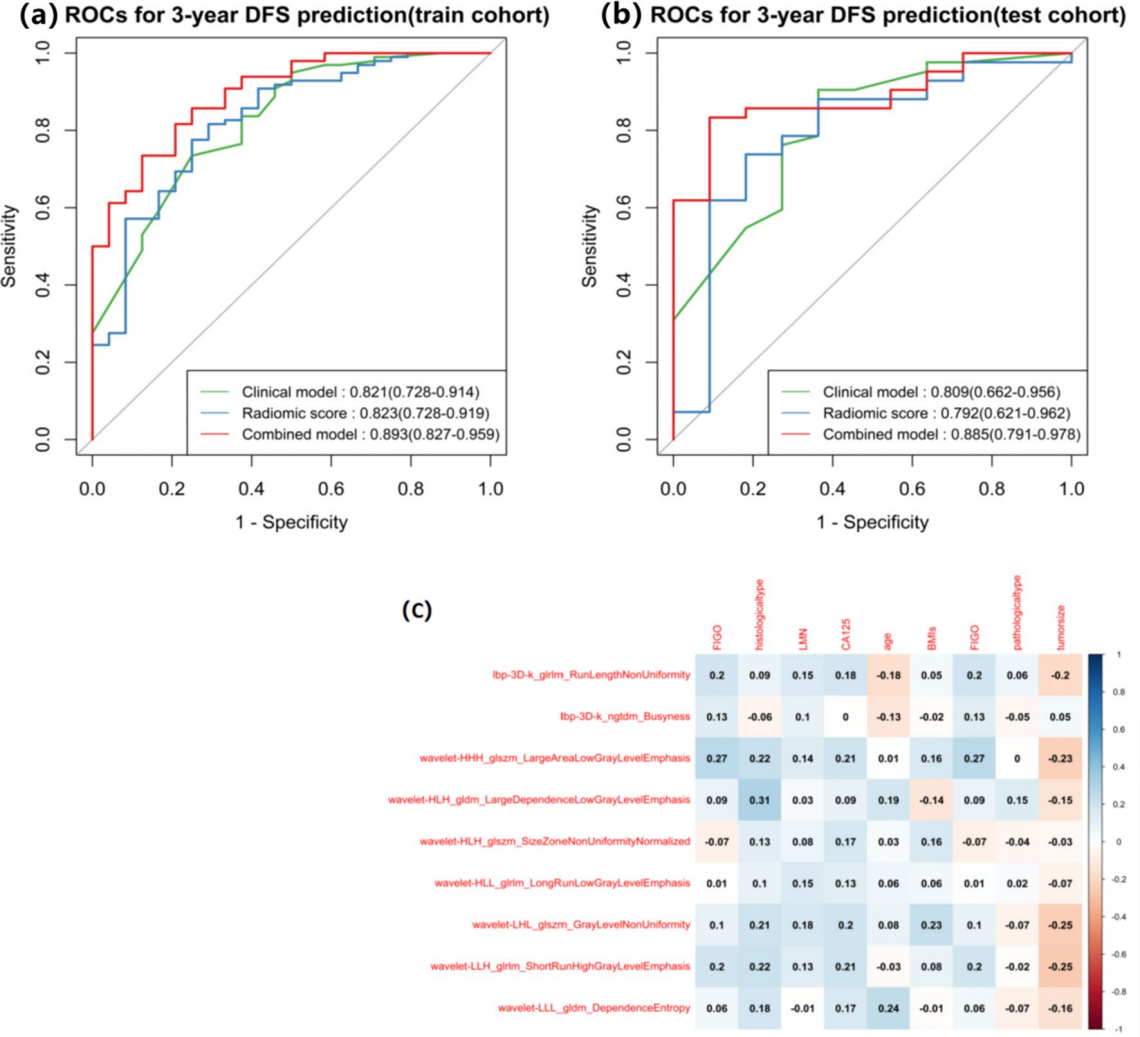
**a** ROC of three models for 3-year DFS in the training cohort. **b** ROC of three models for 3-year DFS in the testing cohort. **c** A heatmap shows the correlations between radiomics features and clinical parameters

A total of 1130 imaging features were extracted with nonzero coefficients from each patient, and nine features were ultimately selected using a LASSO regression model in the training cohort. Figure 2 shows the work flow of feature extraction and the model development. The formula for calculating rad-scores is shown in Eq. 1. We further stratified the patients into high- and low-risk groups according to the rad-score. Patients with higher rad-scores were significantly associated with worse DFS in the training cohort, which was then confirmed in the validation cohort (log-rank test, *P* < 0.0001 and *P* < 0.0001 respectively) (Fig. 3). The rad-score yielded an AUC of 0.823 (95% CI 0.728–0.919), sensitivity of 0.776 (95% CI 0.693–0.858), specificity of 0.750 (95% CI: 0.577–0.923), and accuracy of 0.770 (95% CI 0.768–0.773) in the training cohort, and an AUC of 0.792 (95% CI 0.621–0.962), sensitivity of 0.738 (95% CI 0.605–0.871), specificity of 0.818 (95% CI 0.590–1.046), and accuracy of 0.755 (95% CI 0.748–0.762) in the validation cohort (Table 2, Figs. 4a and b). In addition, the rad-score of each patient is shown in Figs. 3c and d.

1 $$ \begin{aligned} {\text{Rad}} - {\text{score}} & = 0.{1}0{93}*{\text{ "lbp}} - {\text{3D}} - {\text{k}}\_{\text{glrlm}}\_{\text{RunLengthNonUniformity"}} \\ & + 0.{445}0*{\text{" lbp}} - {\text{3D}} - {\text{k}}\_{\text{ngtdm}}\_{\text{Busyness"}} \\ & + 0.{123}0*{\text{"wavelet - HHH}}\_{\text{glszm}}\_{\text{LargeAreaLowGrayLevelEmphasis"}} \\ & + 0.0{681}*{\text{"wavelet - HLH}}\_{\text{gldm}}\_{\text{LargeDependenceLowGrayLevelEmphasis"}} \\ & + 0.{2368}*{\text{"wavelet - HLH}}\_{\text{glszm}}\_{\text{SizeZoneNonUniformityNormalized"}} \\ & + 0.{3325}*{\text{"wavelet - HLL}}\_{\text{glrlm}}\_{\text{LongRunLowGrayLevelEmphasis"}} \\ & + 0.{283}0*{\text{"wavelet - LHL}}\_{\text{glszm}}\_{\text{GrayLevelNonUniformity"}} \\ & + 0.0{163}*{\text{"wavelet - LLH}}\_{\text{glrlm}}\_{\text{ShortRunHighGrayLevelEmphasis"}} \\ & + 0.{2192}*{\text{"wavelet - LLL}}\_{\text{gldm}}\_{\text{DependenceEntropy"}} \\ \end{aligned} $$

**Table 2:** Model performance on predicting DFS

	C-index (95% CI)	AUC (95% CI)	ACC (95% CI)	Sensitivity (95% CI)	Specificity (95% CI)
Clinical model training	0.838 (0.760–0.916)	0.821 (0.728–0.914)	0.738 (0.735–0.741)	0.735 (0.647–0.822)	0.750 (0.577–0.923)
Clinical model validation	0.807 (0.674–0.940)	0.809 (0.662–0.956)	0.849 (0.844–0.854)	0.905 (0.816–0.994)	0.636 (0.352–0.921)
Radiomic score training	0.782 (0.692–0.872)	0.823 (0.728–0.919)	0.770 (0.768–0.773)	0.776 (0.693–0.858)	0.750 (0.577–0.923)
Radiomic score validation	0.778 (0.659–0.896)	0.792 (0.621–0.962)	0.755 (0.748–0.762)	0.738 (0.605–0.871)	0.818 (0.590–1.046)
Combined model training	0.883 (0.830–0.936)	0.893 (0.827–0.959)	0.762 (0.759–0.765)	0.735 (0.647–0.822)	0.875 (0.743–1.007)
Combined model validation	0.883 (0.831–0.936)	0.885 (0.791–0.978)	0.849 (0.844–0.854)	0.833 (0.721–0.946)	0.909 (0.739–1.079)

CI represents confidence interval. C-Index represents Harrell’s concordance index, which measures the performance of the DFS prediction. AUC represents area under the receiver operating characteristic curve, and ACC is accuracy. AUC and ACC evaluate the performance of the 3-year DFS prediction

### Development and validation of prediction models

Clinical model: Univariate analysis showed that FIGO stage (*P* < 0.001), differentiation type (*P* < 0.001), LNM (*P* < 0.001), DMI (*P* = 0.001), CA-125 (*P* < 0.001), and BMI (*P* = 0.010) were significant predictors of DFS. After multivariate analysis, FIGO stage, differentiation type, and CA-125 level (*P* = 0.009, *P* = 0.021, and *P* < 0.001, respectively) were demonstrated as independent predictors of DFS. A clinical model was constructed with the independent predictors, yielding an AUC of 0.821 (95% CI 0.728–0.914) in the training cohort and 0.809 (95% CI 0.662–0.956) in the validation cohort. The results of univariate and multivariate analyses between clinical variables are shown in Supplementary Table.

Combined model: To evaluate the incremental value of the rad-score, a combined model incorporating the rad-score and independent clinical predictors was further developed to predict 3-year DFS. The combined model yielded an AUC of 0.893 (95% CI 0.827–0.959), sensitivity of 0.735 (95% CI 0.647–0.822), specificity of 0.875 (95% CI 0.743–1.007), and accuracy of 0.762 (95% CI 0.759–0.765) in the training cohort, and an AUC of 0.885 (95% CI 0.791–0.978), sensitivity of 0.833 (95% CI 0.721–0.946), specificity of 0.909 (95% CI 0.739–1.079), and accuracy of 0.849 (95% CI 0.844–0.854) in the validation cohort (Table 2, Fig. 3), showing good performance improvement in 3-year DFS estimation when compared with the rad-score alone or clinical model alone. Moreover, the heatmap in Fig. 4c showed that some radiomics features were correlated with clinical parameters (*P* < 0.05 in the cell).

### Nomogram built from the combined model

**Fig. 5:**
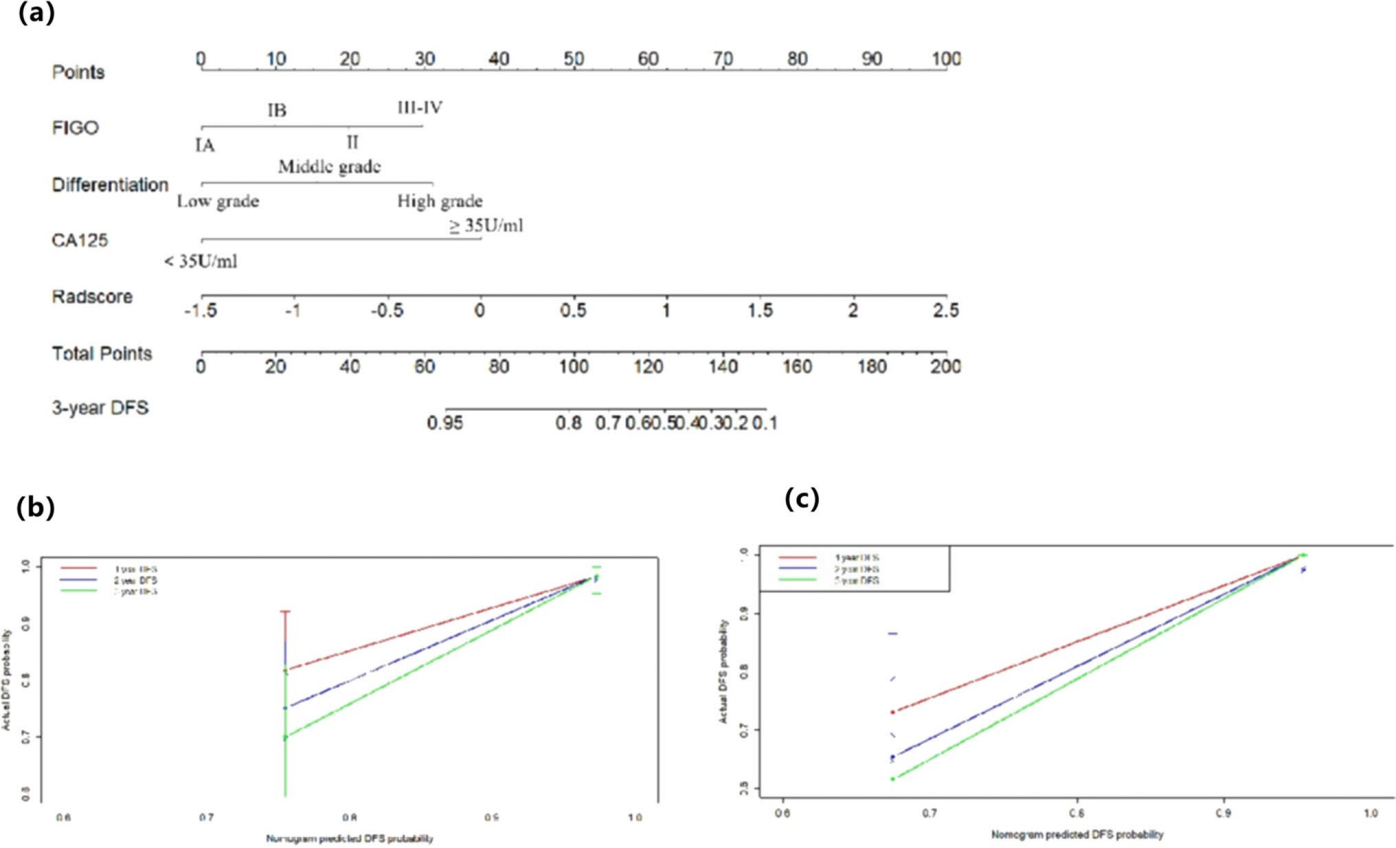
**a** The nomogram was made to predict the risk of DFS in patients with EC. **b** The calibration curves of the nomogram demonstrate the predictive performance for 3-year DFS in the training cohort. **c** The calibration curves of the nomogram demonstrate the predictive performance for 3-year DFS (color green) in the validation cohort

To provide a visualized outcome measure, a nomogram was then built from the combined model (Fig. 5a). Each variable can be proportionally converted into 0 to 100 points by drawing a vertical line upward to the point axis. The total score was then determined by the summary of each variable point, which could evaluate 3-year DFS rate by drawing a line straight down to the outcome axis. According to the nomogram, rad-score has a favorable performance in 3-year DFS prediction. The calibration curves of the nomogram revealed that the prediction values at 3 years for the prognosis for DFS agreed with the actual clinical observation values (Figs. 5b and c).

## Discussion

In the present study, we developed a model that combined FIGO, differentiation, CA-125 level, and significant radiomics features to predict the recurrence risk and achieved a good predictive value, as evidenced by the AUC of 0.893 and 0.885 in the training and validation cohorts, respectively. A visualized nomogram demonstrated that rad-score had favorable predictive performance in DFS prediction.

Radiomics features [18, 21] have been shown to be closely related to genetic and biological characteristics of a tumor, reflecting the texture information of the tumor, which is an important marker of intra-tumor homogeneity. According to some studies, tumors with greater intra-tumoral heterogeneity are correlated with more aggressive behavior such as angiogenesis, proliferation, and metastasis [18, 21–23]. Texture analysis as a post-processing tool may complement the prognostic information obtained from standard imaging.

Concerning the relationship between outcomes in patients with EC and texture features from medical images, Jacob et al. [24] showed that MRI imaging-based texture features were significantly associated with disease-specific survival. Kurtosis in T1c images at filter level 2 (T1c_Kurtosis2) from MRI images was proven by Ytre-Hauge et al. [25] to predict the survival of EC patients independently when adjusted for high-risk status of poor prognosis based on MRI-measured tumor volume. Studies of EC prediction using radiomics from PET are still rare in the literature. The combination of 18F-FDG PET performed in 74 EC patients and machine learning investigated by Nakajo [26] yielded a good result for survival analyses, but it was limited by its small sample size and the fact that it was time consuming. Given this background, it seemed that texture features could be used for risk stratification and prognosis prediction in EC patients.

Considering the increasing use of ultrasound imaging in decision making for EC and satisfactory application of ultrasound radiomics in tumors [18, 19, 27], therefore, we hypothesized that texture analysis on the basis of ultrasound could allow DFS prediction in EC. In our study, nine radiomics features were extracted from ultrasound images. Among these nine parameters, seven wavelet-related features were selected, which were extracted from images decomposed by undecimated 3D wavelet transforms and may not suffer from information loss due to quantization. Fine and coarse textures extracted from the wavelet decomposed images enabled us to design the spatial heterogeneity at multiple scales within tumor regions [28]. This observation was in line with previous studies that used wavelet-based features in the radiomics models [29, 30]. Zhang et al. [29] developed a prediction model for biomarkers of immunophenotyping and survival prognosis based on four features in intrahepatic cholangiocarcinoma patients, three of which were wavelet features. A radiomics study was conducted by Liang et al. [30] for identification of pathological grades in patients with pancreatic neuroendocrine tumors. Seven out of eight features included in the present model were wavelet features. Therefore, these studies along with our results confirmed that wavelet features were significant features in reflecting the biological behavior of tumors. A rad-score was then established based on these nine features, and was demonstrated independently to be associated with DFS and yielded an AUC of 0.823 in the training cohort and 0.792 in the validation cohort, which supported the notion that radiomics has the ability to reflect intra-tumoral heterogeneity.

In addition, the rad-score calculated in our study allowed us to stratify patients into high- and low-risk groups and helped us identify EC patients with worse DFS for whom adjuvant treatment and a close follow-up schedule were needed. Furthermore, patients with EC at low risk could avoid over-treatment. Our study suggested that higher rad- scores were associated with a poor prognosis. These findings will enable clinicians to tailor individual treatment on the basis of the clinical and radiomics features for high risk and low-risk patients with EC.

In our study, FIGO stage, CA-125, and differentiation were demonstrated to be significant independent clinical risk predictors and were incorporated into our nomogram. Previous studies [31] have shown that FIGO stage is the most commonly used system to predict EC outcomes. EC patients in stage I-II may have favorable outcomes, whereas those in stage III or IV may have adverse DFS. CA-125 level is known to be an independent risk factor for predicting hematogenous EC recurrence [32], and CA-125 levels are significantly higher in patients with advanced FIGO stage [33]. Our study demonstrated that differentiation was a significant independent prognostic factor for DFS, which was consistent with previous studies [34]. We ultimately developed a combined model that integrated ultrasound radiomics signatures with clinical parameters to further improve the predictive accuracy for DFS, which yielded a favorable outcome (AUC of 0.893 and 0.885 in the training and validation cohorts, respectively). Furthermore, the radiomics signatures also yielded comparable results concerning clinical parameters in our study.

Our study had several limitations. First, it was a retrospective study in which most of the recorded images were discrete three-dimensional images. Only a few images were analyzed per patient. In some cases, the most representative part of the tumor may not have been captured. Second, the sample size was relatively small since patients without ultrasound images in our hospital before endometrial suction biopsy, dilatation and curettage (D&C), or hysteroscopy were excluded. Selection bias may occur when such strict inclusion criteria are used. Third, our models were performed in a single hospital and without external validation, which could have reduced the confirmation strength of the model accuracy. Future studies involving a larger sample should be carried out, and multicenter external validation will be needed.

## Conclusion

In conclusion, patients with higher rad-scores were significantly associated with worse DFS. The nomogram that incorporated the radiomics signature and clinical parameters showed good predictive performance and potential to predict DFS in patients with EC.

### Supplementary Information

Below is the link to the electronic supplementary material.

Supplementary file1 (DOCX 14 kb)

## Data Availability

The data underlying this article will be shared on reasonable request to the corresponding author.
