# Correction: An ultrasound-based radiomics model for survival prediction in patients with endometrial cancer

**DOI:** 10.1007/s10396-024-01438-8

**Published:** 2024-03-21

**Authors:** Xiao-wan Huang, Jie Ding, Ru-ru Zheng, Jia-yao Ma, Meng-ting Cai, Martin Powell, Feng Lin, Yun-jun Yang, Chu Jin

**Affiliations:** 1 Department of Gynecology, The First Affiliated Hospital of Wenzhou Medical University, Wenzhou, 325000 People’s Republic of China; 2 Wenzhou Medical University Renji College, University Town, Chashan, Wenzhou, 325000 People’s Republic of China; 3 Department of Ultrasound Imaging, Yueqing Hospital of Wenzhou Medical University, Wenzhou, 325015 People’s Republic of China; 4 Department of Radiology, The First Affiliated Hospital of Wenzhou Medical University, Wenzhou, 325000 People’s Republic of China; 5 Nottingham Treatment Centre, Nottingham University Affiliated Hospital, Nottingham, NG7 2FT UK

**Correction: Journal of Medical Ultrasonics (2023) 50(4):501–510** 10.1007/s10396-023-01331-w

The article “An ultrasound‑based radiomics model for survival prediction in patients with endometrial cancer”, written by Xiao‑wan Huang, Jie Ding, Ru‑ru Zheng, Jia‑yao Ma, Meng‑ting Cai, Martin Powell, Feng Lin, Yun‑jun Yang and Chu Jin, was originally published Online First without Open Access. After publication in volume 50, issue 4, pages 501–510 the author decided to opt for Open Choice and to make the article an Open Access publication. Therefore, the copyright of the article has been changed to © The Author(s) 2023 and the article is forthwith distributed under the terms of the Creative Commons Attribution 4.0 International License, which permits use, sharing, adaptation, distribution and reproduction in any medium or format, as long as you give appropriate credit to the original author(s) and the source, provide a link to the Creative Commons licence, and indicate if changes were made. The images or other third party material in this article are included in the article’s Creative Commons licence, unless indicated otherwise in a credit line to the material. If material is not included in the article’s Creative Commons licence and your intended use is not permitted by statutory regulation or exceeds the permitted use, you will need to obtain permission directly from the copyright holder. To view a copy of this licence, visit http://creativecommons.org/licenses/by/4.0.

The original article has been corrected.

