# Supplementary material for: An ultrasound-based radiomics model for survival prediction in patients with endometrial cancer
Source: J Med Ultrason (2001). 2023 Jun 13;50(4):501–10. doi: 10.1007/s10396-023-01331-w (PMC10955020; doi:10.1007/s10396-023-01331-w)
Supplement: Supplementary file 1 — Supplementary file1 (DOCX 14 kb) [file 10396_2023_1331_MOESM1_ESM.docx]

**Supplementary Table**

Univariable and multivariable analyses between clinical variables

| Variable | Univariable Analysis | | Multivariable Analysis | |
| --- | --- | --- | --- | --- |
|  | OR (95% CI) | p-value | OR (95% CI) | p-value |
| Age (years) |  |  |  |  |
| ＜60 | 1 |  |  |  |
| ≥60 | 1.706 (0.878-3.311) | 0.115 |  |  |
| Menopause status |  |  |  |  |
| No | 1 |  |  |  |
| Yes | 1.437 (0.655-3.153) | 0.366 |  |  |
| BMI | 2.374 (1.233-4.571) | 0.010 | 2.418 (1.23-4.753) | 0.051 |
| FIGO |  | ＜0.001 |  | 0.009 |
| IA | 1 |  | 1 |  |
| IB | 2.623 (0.878-7.833) | 0.084 | 1.575 (0.513-4.838) | 0.427 |
| II | 1.471 (0.453-4.777) | 0.521 | 1.15 (0.352-3.75) | 0.817 |
| III-IV | 9.676 (4.321-21.667) | ＜0.001 | 5.504 (1.979-15.305) | 0.001 |
| Pathological type |  |  |  |  |
| Endometrioid adenocarcinoma | 1 |  |  |  |
| Non-endometrioid adenocarcinoma | 1.969 (0.947-4.092) | 0.070 |  |  |
| Differentiation |  | ＜0.001 |  | 0.021 |
| Low grade | 1 |  | 1 |  |
| Middle grade | 5.100 (1.166-22.306) | 0.030 | 2.252 (0.491-10.331) | 0.296 |
| High grade | 9.378 (2.183-40.283) | ＜0.001 | 5.007 (1.117-22.456) | 0.035 |
| Tumor size |  |  |  |  |
| ＜2cm | 1 |  |  |  |
| ≥2cm | 0.440 (0.135-1.434) | 0.173 |  |  |
| Lymph node metastasis（LNM） |  |  |  |  |
| Non-metastasis | 1 |  | 1 |  |
| Metastasis | 7.304 (3.608-14.785) | ＜0.001 | 0.752 (0.261-2.165) | 0.597 |
| Lymphovascular space invasion (LVSI) |  |  |  |  |
| Non-LVSI | 1 |  |  |  |
| LVSI | 1.884 (0.8254.304-) | 0.133 |  |  |
| Depth of myometrial invasion(DMI) |  |  |  |  |
| ＜1/2 | 1 |  | 1 |  |
| ≥1/2 | 3.019 (1.567-5.814) | 0.001 | 0.95 (0.317-2.849) | 0.927 |
| CA125 |  |  |  |  |
| ＜35 U/ml | 1 |  | 1 |  |
| ≥35 U/ml | 7.462 (3.862-14.416) | ＜0.001 | 4.224 (1.985-8.992) | ＜0.001 |
